# Negative correlation between ACE2 gene expression levels and loss of taste in a cohort of COVID-19 hospitalized patients: New clues to long-term cognitive disorders

**DOI:** 10.3389/fcimb.2022.905757

**Published:** 2022-09-29

**Authors:** Isabela Braga-Paz, João Locke Ferreira de Araújo, Hugo José Alves, Renata Eliane de Ávila, Gustavo Gomes Resende, Mauro Martins Teixeira, Renato Santana de Aguiar, Renan Pedra de Souza, Diana Bahia

**Affiliations:** ^1^ Departamento de Genética, Ecologia e Evolução, Instituto de Ciências Biológicas, Universidade Federal de Minas Gerais, Universidade Federal de Minas Gerais (UFMG), Belo Horizonte, Brazil; ^2^ Hospital Eduardo de Menezes, Belo Horizonte, Brazil; ^3^ Hospital das Clínicas, Universidade Federal de Minas Gerais (HC-UFMG/EBSERH), Belo Horizonte, Brazil; ^4^ Departamento de Bioquimica e imunologia, Instituto de Ciências Biológicas, Universidade Federal de Minas Gerais, Universidade Federal de Minas Gerais (UFMG), Belo Horizonte, Brazil; ^5^ Instituto D’Or de Pesquisa e Ensino, Instituto D'OR (IDOR), Rio de Janeiro, Brazil

**Keywords:** COVID-19, severe COVID-19, anosmia, ageusia, genetic association, quantitative trait, long-COVID syndrome, cognitive dysfunction

## Abstract

In early 2020, one of the most prevalent symptoms of SARS-CoV-2 infection was the loss of smell (anosmia), found in 60-70% of all cases. Anosmia used to occur early, concomitantly with other symptoms, and often persisted after recovery for an extended period, sometimes for months. In addition to smell disturbance, COVID-19 has also been associated with loss of taste (ageusia). The latest research suggests that SARS-CoV-2 could spread from the respiratory system to the brain through receptors in sustentacular cells localized to the olfactory epithelium. The virus invades human cells *via* the obligatory receptor, angiotensin-converting enzyme II (ACE2), and a priming protease, TMPRSS2, facilitating viral penetration. There is an abundant expression of both *ACE2* and *TMPRSS2* in sustentacular cells. In this study, we evaluated 102 COVID-19 hospitalized patients, of which 17.60% presented anosmia and 9.80% ageusia. *ACE1*, *ACE2*, and *TMPRSS2* gene expression levels in nasopharyngeal tissue were obtained by RT-qPCR and measured using ΔCT analysis. ACE1 *Alu*287bp association was also evaluated. Logistic regression models were generated to estimate the effects of variables on ageusia and anosmia Association of *ACE2* expression levels with ageusia. was observed (OR: 1.35; 95% CI: 1.098-1.775); however, no association was observed between *TMPRSS2* and *ACE1* expression levels and ageusia. No association was observed among the three genes and anosmia, and the *Alu*287bp polymorphism was not associated with any of the outcomes. Lastly, we discuss whetherthere is a bridge linking these initial symptoms, including molecular factors, to long-term COVID-19 health consequences such as cognitive dysfunctions.

## 1 Introduction

Severe Acute Respiratory Syndrome Coronavirus 2 (SARS-CoV-2) is the virus responsible for causing coronavirus disease 2019 (COVID-19). Among the most common COVID-19 symptoms are fever, cough, fatigue, and anosmia (loss of smell) with or without ageusia (loss of taste) in mild to moderate cases (Huang et al., 2020; Aghagoli et al., 2021).

The SARS-CoV-2 virus can lead to neurologic dysfunction by both direct and indirect mechanisms, including damage to olfactory sensory neurons, impairing the brain’s olfactory perception center, and post-viral anosmia. The infection can also lead to a cytokine storm that can damage the blood-brain barrier and disrupt the normal functioning of the Central Nervous System (CNS). Reports of prothrombotic status were also associated with COVID-19, leading to the obstruction of cerebral vessels and causing ischemic CNS lesions (Yazdanpanah et al., 2020; Aghagoli et al., 2021).

Complete or partial loss of smell – leading to a decreased sense of taste- may be related to olfactory disorders, head trauma, or viral infections. Although the mechanism of how viral infection causes anosmia is unclear, it is believed to involve the destruction of the olfactory neuroepithelium or transmission of pathogens directly through the olfactory nerve (Whitcroft and Hummel, 2020; Meng and Pan, 2021).

In patients affected by SARS-CoV-2 in 2020, reports of anosmia were observed in the acute phase of the disease even in the absence of other symptoms, thus becoming a hallmark symptom of COVID-19 (Hopkins et al., 2020; Lechien et al., 2020; Whitcroft and Hummel, 2020; Glezer et al., 2021; Meng and Pan, 2021). In most cases, the sense of smell recovered in about two weeks or after other symptoms improved. However, in some patients, the recovery of smell and taste took longer, a phenomenon that may be associated with the effect of the virus on the CNS and warranted further investigation (Hopkins et al., 2020; Lechien et al., 2020; Glezer et al., 2021). Many patients are now experiencing long-term health consequences called long-COVID syndrome. As the pandemic progressed, SARS-CoV-2 variants of concern (VOCs) emerged (Mistry et al., 2021; Zawilska et al., 2021). These variants have been linked to higher mortality rates, clinical complications, and increased viral transmission rates (Choi and Smith, 2021; Raman et al., 2021). In addition, variability in COVID-19 symptomatology was observed with different variants (Pedro et al., 2021).

The Spike protein (S) of SARS-CoV-2 is a viral envelope glycoprotein responsible for binding to angiotensin-converter enzyme 2 (ACE2) after its cleavage at sites S1/S2 by type II transmembrane serine protease (TMPRSS2) (Johnson et al., 2021; Meinhardt et al., 2021). The SARS-CoV-2 virus invades human cells *via* the obligatory ACE2 receptor, and TMPRSS2 further facilitates viral uptake. Co-expression of *ACE2* and *TMPRSS2* was observed in ciliated epithelial cells, nasopharyngeal tissue, and on the surface of oligodendrocytes (Sardu et al., 2020; Martínez-Gómez et al., 2022). The ratio between *ACE1* and *ACE2* has been implicated in the pathogenesis of respiratory diseases, and the functional polymorphisms of these genes have been associated with an increased risk of lung and cardiovascular diseases. Therefore, these genes may contribute to COVID-19 outcomes (Choudhary et al., 2020; Galisa et al., 2021; Martínez-Gómez et al., 2022).

The *ACE1* gene modulates *ACE2* expression with increased levels of angiotensin II. Both are part of the renin-angiotensin-aldosterone system (RAAS) (D’ardes et al., 2020; Saad et al., 2021). A 287 bps *Alu* sequence rs4646994, repeated insertion/deletion (I/D) (indel) in intron 16, has been described in the *ACE1* gene, which leads to an expression modulation of this gene (Castellon and Hamdi, 2007; de Araújo et al., 2022). A recent study has shown that an *Alu* homozygous deletion (D/D) is associated with the progression of severe COVID-19 (de Araújo et al., 2022). However, studies are still required to evaluate the role of the genetic polymorphism in COVID-19 outcomes.

This study investigates the gene expression of potential viral targets in the olfactory epithelium in nasal swab samples obtained from 102 hospitalized patients with COVID-19 between April and September 2020. We explore *ACE1*, *ACE2*, and *TMPRSS2* expression in these patients and the relationship of these genes with the onset of symptoms such as anosmia and ageusia. Information on anosmia and ageusia was collected through the hospitalized patient’s report. Moreover, we discuss whether the prevalence of these symptoms months after infection could lead to neurological problems or cognitive dysfunction such as short-term memory loss, difficulty concentrating, mental confusion, and imbalance.

## 2 Methods

### 2.1 Patients

Samples were collected from 102 COVID-19 positive patients confirmed by qPCR and were admitted to the Eduardo de Menezes hospital in Belo Horizonte, Minas Gerais, Brazil, between April and September 2020, before the appearance of SARS-CoV-2 variants of concern. Exclusion criteria were cancer, autoimmune diseases, and pregnancy. Smoking status was also reported. All participants gave informed consent (CAAE 32224420.3.0000.0008 and CAAE 31462820.3.0000.5149). When patients could not consent due to hospitalization, informed consent was obtained by a legal guardian. RT-qPCR confirmed COVID-19 diagnosis. The clinical data of the patients used in the study was obtained from hospital records. Patients self-reported anosmia and ageusia. Information about comorbidities is based on the medical history of each hospitalized patient. Nasopharyngeal swab samples were collected in a viral transport medium (DMEM or PBS) and stored at -80°C until extraction. The collection of biological material was performed on the first day of hospitalization of the patients.

### 2.2 Expression analysis

RNA extraction was performed using a Quick-RNA Viral kit (Zymo Research, CA, USA). Samples were treated with TURBO DNA-free kit (Thermo Fisher Scientific, MA, USA) when GoTaq SYBR green qPCR assay was employed. cDNA was generated using the High-Capacity cDNA Reverse Transcription Kit (Thermo Fisher Scientific, MA, USA). All above reactions were conducted following the manufacturer’s instructions. *ACE1*, *ACE2*, *TMPRSS2*, and *B2M* (reference gene) gene expression levels were evaluated using the GoTaq Probe qPCR System (Promega, WI, USA). The specific probes (Integrated DNA Technologies, NJ, USA) used and designed for exon-exon junctions were: Hs.PT.58.19167084, Hs.PT.58.27645939, HS.PT.58.39738666, and Hs.PT.39a.22214847, respectively. ΔCt was calculated by subtracting the cycle threshold (Ct) of the gene of interest from the reference gene (B2M) Ct.

### 2.3 *Alu*287bp polymorphism (rs4646994) genotyping

For the genotyping of the rs4646994 polymorphism, the FastStart Universal SYBR Green Master Kit (Promega, WI, USA) was used to yield a final volume of 20 µL using three different primers: 5’CATCCTTTCTCCCATTTCTC3’ (Primer1, Forward); 5’TGGGATTACAGGCGTGATACAG3’ (Primer 2, Forward, internal); and 5’ATTTCAGAGCTGGAATAAAATT3’ (Primer 3, Reverse). Primer stock was resuspended and diluted to a working solution of 10 µM. The concentration of primers 1 and 3 is 20 pmol, and the concentration of primer 2 is 40 pmol. The generated fragment sizes were 65bp (Insertion) and 84bp (deletion) and visualized on a 3% agarose gel. Ten percent of the sample was randomly genotyped twice to attest to the genotyping quality. The agreement level was 100%.

### 2.4 Statistical analysis

All analyses of this study were performed in R version 4.0.2, considering a significance level of 0.05. The verification of the Hardy-Weinberg equilibrium (HWE) was performed using the SNPassoc package. Allelic and genotypic frequency calculations were performed. Logistic regression models were generated to estimate the individual effects of the variables age, sex, RS4646994 polymorphism, and gene expression levels for the Anosmia and Ageusia outcomes.

A logistic regression model was generated to investigate association between clinical and molecular data. We used Ct data of the N gene of SARS-CoV-2 to evaluate whether there was a difference between the viral loads of patients with COVID-19 experiencing anosmia and ageusia vs. those without anosmia and ageusia. Ct values from RNase P (Ribonuclease P) were used for normalization. The results were reported in **Table 2** as mean, standard deviation (sd), odds ratio (OR), and confidence interval (CI).

## 3 Results

Only one patient did not require respiratory support. There were 54 women and 48 men, and the mean duration of hospitalization was 10.2 ± 6.5 days. The death rate was 13.7%, and the mean age of these patients was 55.11 ± 4.6 years. About 25% of patients had diabetes, 55.9% were hypertensive, 13.7% had asthma, and 20.6% were smokers (**Table 1**).

**Table 1:** Demographic and clinical characteristics of the study sample (n = 102).

Characteristics	Value
Age, mean (sd)	55.1 (14.6)
Hospitalization days, mean (sd)	10.2 (6.5)
Female Sex, N (%)	54 (52.9%)
Death, N (%)	14 (13.7%)
Anosmia, N (%)	18 (17.6%)
Ageusia, N (%)	10 (9.8%)
Respiratory support, N (%)	101 (99%)
Diabetes, N (%)	26 (25.5%)
Obesity N (%)	28 (27.7%)
Smoking N (%)	21 (20.6%)
Hypertension N (%)	57 (55.9%)
Asthma N (%)	14 (13.7%)

sd, standard deviation; No, number; clinical and demographic data from a sample of Covid-19 positive patients admitted to the Eduardo de Menezes hospital, Belo Horizonte, MG. Age, sex, hospitalization days, death and main symptoms.

Hardy Weinberg equilibrium (HWE) analysis was performed separately for case and control groups for the frequency of the *Alu*287bp polymorphism. Anosmia and Ageusia were tested. Both cases were in HWE for the outcome of anosmia and ageusia (p=0.624 and p=0.219, respectively). However, controls for anosmia and ageusia were out of HWE (p=0.021 and p=0.028, respectively). Two individuals could not have their genotypes evaluated (**Table 2**).

**Table 2:** Genotypic and allelic absolute values for the rs4646994 polymorphism.

Sample		Genotype					p value
	n	DD	DI	II	D	I	
**Anosmia case**	18	5	11	2	21	15	0.624
**Anosmia control**	82	35	29	18	99	65	0.021
**Ageusia case**	10	3	7	0	13	7	0.219
**Ageusia control**	90	37	33	20	107	73	0.028

Genotypic and allelic absolute values for the rs4646994 polymorphism for case and control groups in two studies: Chance of anosmia and ageusia. Hardy Weinberg equilibrium performed for case and control separately. Yes: case; No: control.

No difference was observed when the variables age and sex were tested in the logistic model for anosmia. Similarly, there were no differences in *ACE1* gene expression in the anosmia. No association was observed between the manifestation of anosmia and the *ACE2* and *TMPRSS2* expression levels (p=0.063 and p=0.068, respectively) (**Table 3**). No association was observed for the *Alu*287bp polymorphism, testing the codominance, D allele dominance, and I allele dominance models (**Table 3**).

**Table 3:** Logistic regression model for anosmia and ageusia outcomes.

Outcome	Variable	Case		Control		p value	OR CI (95%)
		N	mean (sd) or %	N	mean (sd) or %		
Anosmia	Age	18	55.44 (10.33)	84	54.04 (15.43)	0.916	–
	*ACE1* ΔCT	7	11.17 (5.25)	75	10.77 (3.85)	0.835	–
	*ACE2* ΔCT	9	15.09 (5.82)	79	13.59 (4.75)	0.063	
	*TMPRSS2* ΔCT	9	13.11 (4.99)	79	8.17 (4.59)	0.068	
	Sex (Male)	7	0.39	41	0.49	0.446	
	rs4646994 Codominance DD	5	0.27	35	0.43	–	–
	rs4646994 Codominance DI	11	0.61	29	0.35	0.101	–
	rs4646994 Codominance II	2	0.12	18	0.22	0.777	–
	rs4646994 I allele dominance (DI + II)	13	0.73	47	0.57	0.248	–
	rs4646994 D allele dominance (DD + DI)	16	0.88	64	0.78	0.308	–
Ageusia	Age	10	54.80 (9.41)	92	55.15 (15.11)	0.942	
	*ACE1* ΔCT	7	11.14 (6.09)	75	10.81 (3.83)	0.788	
	*ACE2* ΔCT	9	17.16 (4.41)	79	13.37 (4.93)	0.012	1.35 (1.098-1.775)
	*TMPRSS2* ΔCT	9	13.63 (5.63)	79	8.58 (4.67)	0.074	
	Sex (Male)	4	0.4	44	0.48	0.639	–
	rs4646994 Codominance DD	3	0.3	37	0.41	–	–
	rs4646994 Codominance DI	7	0.7	33	0.37	0.188	–
	rs4646994 Codominance II	0	0	20	0.22	0.994	–
	rs4646994 I allele dominance (DI + II)	7	0.7	53	0.59	0.5	–
	rs4646994 D allele dominance (DD + DI)	10	1	70	0.78	0.991	–

sd, standard deviation; OR, Odds ratio; CI, confidence interval; ΔCT, cycle threshold rate; exploring the variables age, sex rs4646994 polymorphism and ACE1, ACE2 and TMPRSS2 genes expression levels for the presence (yes/case) or absence (no/control) of anosmia or ageusia. The variable “DD +DI” corresponds to the dominance of the D allele and the variable “DI +II” corresponds to the dominance of the I allele.

Age and sex were also not significant when tested in the logistic model for ageusia. There was no difference in *ACE1* gene expression in the ageusia. An association was observed between decreased *ACE2* expression levels and ageusia (OR:1.35; 95%CI: 1.09-1.77). No association for manifestation of ageusia was detected with low *TMPRSS2* gene expression levels (p=0.074) (**Table 3**). No association was observed for the *Alu*287bp polymorphism, testing the codominance, D allele dominance, and I allele dominance models (**Table 3**).

No differences were observed for viral load between patients with anosmia and ageusia and the respective controls (p=0.395 and p=0.931, respectively).

## 4 Discussion

Herein, we evaluated the association of *ACE1*, *ACE2*, and *TMPRSS2* expression with the presence of anosmia and/or ageusia. There was a negative correlation between *ACE2* and ageusia in a sample of hospitalized COVID-19 positive patients. Furthermore, no association was observed between expression of *ACE1* and *TMPRSS2* and the manifestation of ageusia. No association was observed for the three genes and anosmia. In addition, no association was found for the *ALU287pb* polymorphism with the presence of anosmia and/or ageusia,

*ACE2* and *TMPRSS2* actively participate in the SARS-Cov-2 infectious process. Some studies have already demonstrated the association of differentiated levels of expression of these genes in the prognosis of COVID-19 (Sardu et al., 2020). For example, the ACE2/TMPRSS2 ratio was associated with the need for respiratory support (Rossi et al., 2021). Although high *ACE2* and *TMPRSS2* expression levels are associated with a severe prognosis of COVID-19, in this study, we evaluated specific clinical symptoms of the disease. Anosmia and ageusia have been observed more frequently in patients with a mild outcome and lower frequency in those with severe disease (Lechien et al., 2020; Aghagoli et al., 2021). The sample used in this study was obtained from patients hospitalized with severe clinical symptoms. In these samples, we found that low *ACE2* gene expression levels correlated with ageusia.

In addition, both genes are highly expressed on the surface of oligodendrocytes, which could explain the proliferation of the virus in nerve tissue cells and subsequent neurological damage and sequelae. *ACE2* is abundantly expressed in the respiratory epithelium and in cells of the oral mucosa tissue, mainly in epithelial cells of the oral mucosa, lips, and tongue, these sites being more susceptible to SARS-CoV-2 invasion compared to other oral anatomical sites, and direct damage to the oral epithelium and the neuroinvasive nature of the virus can lead to taste disturbances (Zhong et al., 2020; Farid et al., 2022). Thus, *ACE2* and *TMPRSS2* expression may be key factors in unveiling neurocognitive consequences of long-COVID-19 syndrome (Huang et al., 2020; Aghagoli et al., 2021).

In animal models, the SARS-CoV-2 virus down-regulates *ACE2* expression leading to an imbalance in the *ACE1/ACE2* cerebrovascular control, resulting in an ACE1 signal mismatch, excessive vasoconstriction, or brain autoregulation disorders (Aghagoli et al., 2021). A recent meta-analysis showed that the 287bp deletion in the *ACE1* gene is associated with the prognosis of severe COVID-19 (de Araújo et al., 2022). Nonetheless, the authors have not evaluated long-term health disorders yet. In this study, age and sex did not correlate with onset of anosmia and ageusia. However, age and sex remain important variables in the prognosis of COVID-19. Several studies have shown higher frequencies of disease severity and death in elderly individuals (Izcovich et al., 2020; Izurieta et al., 2020). Sex has also been a factor in COVID-19 prognosis and mortality, mainly affecting male individuals (Ghamrawi and Gunaratne, 2020; Izurieta et al., 2020). A prospective observational cohort study of COVID-19 analyzed 4,182 individuals who self-reported their symptoms in the “COVID Symptom Study app”- a mobile health app developed by Zoe Global (Sudre et al., 2021). In the study, those with long-term COVID were mostly individuals over 70 and more likely to be female.

Currently, evidence indicates that SARS-CoV-2 does not affect only the respiratory tract but also the CNS, which has resulted in symptoms such as anosmia, ageusia, headache, fatigue, nausea, vomiting, acute cerebrovascular disease, and altered consciousness (Conde Cardona et al., 2020; Huang et al., 2020; Meinhardt et al., 2021). Anosmia rates are lower among patients infected by the Gamma and Omicron strains than the original strain. Besides that, no differences were observed between the original strain and the Delta variant (Cardoso et al., 2022).

Here we started to raise the hypothesis and discuss whether there is a bridge linking initial symptoms, such as anosmia and ageusia, modulation of molecular factors expression, to long-COVID, which is the long-term COVID-19 health consequences experienced by an increasing number of patients. There are five types of specialized cells in the olfactory epithelium, namely the sustentacular cells, basal cells, microvillar cells, the olfactory sensory neurons (OSNs), and the ductal cells of Bowman’s glands (Riel et al., 2015; Ahmed et al., 2022). The expression of the input factors of SARS-CoV-2 in the olfactory epithelium, resulting in loss of OSNs, may lead to anosmia. Smell and taste disorders are related to our general and mental health and can be early indicators of CNS compromises (Brechbühl et al., 2021; Glezer et al., 2021).

A study investigating viral entry sites and sensory symptoms in mice found that the virus can target chemosensory cells of the gustatory and olfactory systems (Brechbühl et al., 2021). Although the direct action of the virus on these cells is not fully elucidated, mechanisms of cytopathic destruction impact sense of smell and taste, and this impairment could directly contribute to anosmia and ageusia in the long term (Cazzolla et al., 2020; Brechbühl et al., 2021; Muus et al., 2021). Herein, we have not observed differences in viral load between patients with anosmia and ageusia and the respective controls, suggesting that other mechanisms or factors may be involved in developing long-COVID syndrome.

There are several symptoms related to COVID-associated cognitive impairment, which are persistent and worsen the quality of life of COVID-19 survivors (Becker, 2021; Nasserie et al., 2021). Long-COVID syndrome is associated with impaired concentration, information processing, memory, executive functioning, anxiety, depression, panic syndrome, sleep disorder, and fatigue. COVID-dependent cognitive impairments contribute substantially to reduced global functioning. Long-COVID syndrome has been described to typically last up to 6 to 12 months but can last indefinitely in some individuals (Orrù et al., 2021; Titze-de-Almeida et al., 2022).

In a study (de Paula et al., 2022) performed with 195 individuals with mild COVID-19, 26% had a visuoconstructive deficiency, a typical early manifestation of Alzheimer’s disease. There was also increased expression of 11 plasma biomarkers, including chemokines and growth factors. These biomarkers showed a strong positive correlation with the degree of cognitive impairment. The disability has persisted for up to a year after diagnosis. People who had mild COVID-19 and were not hospitalized in the two to ten months before enrollment, and without comorbidities were included. Most of the volunteers are health professionals who work in the hospital region and learned about the study through advertisements made by the institutions. The mean age of the cohort was 38 years (de Paula et al., 2022).

A group from England recently published a robust study involving 785 participants (aged 51–81) with COVID (Douaud et al., 2022), including patients with only mild COVID-19 symptoms. Limbic brain imaging was obtained twice and compared to 384 non-infected controls. They identified a consistent spatial pattern of longitudinal anomalies in limbic brain regions forming a mainly olfactory network, including a greater reduction in global brain size.

The authors hypothesized that the results could represent the hallmarks of the degenerative spread of the disease *via* olfactory pathways, neuroinflammatory events, or loss of sensory input due to anosmia and suggested longer follow-up periods to evaluate these effects. Brain atrophy (due to the loss of input received from the nose) is the only plausible explanation for linking anosmia symptoms in COVID-19 to brain/cognitive symptoms seen in long-COVID Syndrome (Marshall, 2022).

Our group hypothesized that, in addition to viral entry into cells in the CNS, there are signaling pathways activated during the COVID-19 that disrupt the brain homeostasis, thereby leading to long term neurocognitive consequences. As such, anosmia and ageusia could be the early events heralding the neurocognitive changes of long-COVID-19 syndrome. A better understanding of anosmia and ageusia may improve our knowledge of long-lasting cognitive disorders, including long-COVID-19. Future studies will investigate the relative expression of key olfactory and sustentacular cell marker genes in samples collected from post-COVID-19 patients (six months to one year after contracting COVID-19) with or without anosmia and/or ageusia, using neuropsychological tests (de Paula et al., 2022).

To the best of our knowledge, this is the first study utilizing gene expression in nasopharyngeal swab samples of COVID-19 patients to identify expression differences associated with anosmia and ageusia. Future directions include testing a higher number of putative target genes with longer symptom follow-up to obtain better associations between expression differences and long-COVID-19 symptoms and duration. A better understanding of the genes modulated in association with initial anosmia and ageusia may bright light on the signaling pathway alterations that are associated with neurologic symptoms of COVID-19 and the development of long-COVID-19 syndrome and lay the groundwork for pharmacologic therapies that interfere with pathologic signaling changes.

## Data availability statement

The original contributions presented in the study are included in the article. Further inquiries can be directed to the corresponding authors.

## Ethics statement

The studies involving human participants were reviewed and approved by Certificado de Apresentação de Apreciação Ética: CAAE 32224420.3.0000.0008 and CAAE 31462820.3.0000.5149. The patients/participants provided their written informed consent to participate in this study.

## Author contributions

DB and RS conceived the study. IB-P, JA, REA, RS, and DB designed the experiments. IB-P, JA, HA, and RS performed the experiments. IB-P, JA, RSA, RS, and DB interpreted the results and analyzed the data. RSA, RS, and DB contributed with material and analysis tools. REA, GR and MT were responsible for subject’s recruitment and clinical follow-up. IB-P, JA, and DB wrote the manuscript. All authors read and approved the final manuscript.

## Funding

The authors wish to thank the following funding sources:Rede Corona-ômica BR MCTI/FINEP affiliated to RedeVırus/ ´MCTI (FINEP 01.20.0029.000462/20, CNPq 404096/2020-4),MEC/CAPES (14/2020–23072.211119/2020-10), FINEP (0494/20 01.20.0026.00 and UFMG-NB3 1139/20), FAPEMIG (RPS:APQ-00475-20), FAPERJ (RSA 202.922/2018) and CAPES(88881.506612/2020-0). RPS, RSA, and DB are recipients of CNPq Fellowships.

## Acknowledgments

The authors also thank BioMed Proofreading LLC for the English language editing of this manuscript.

## Conflict of interest

The authors declare that the research was conducted in the absence of any commercial or financial relationships that could be construed as a potential conflict of interest.

## Publisher’s note

All claims expressed in this article are solely those of the authors and do not necessarily represent those of their affiliated organizations, or those of the publisher, the editors and the reviewers. Any product that may be evaluated in this article, or claim that may be made by its manufacturer, is not guaranteed or endorsed by the publisher.
